# Contingency planning for cancer care in low- and middle-income countries during the COVID-19 pandemic: a rapid assessment for future disaster resilience

**DOI:** 10.3332/ecancer.2022.1339

**Published:** 2022-01-06

**Authors:** Soo-Peng Teoh, Yee-Yin Hoo, Raul Murillo, María Zuluaga, Audrey Tsunoda, Dorothy Lombe, Richard Sullivan, Nirmala Bhoo-Pathy

**Affiliations:** 1 Centre for Epidemiology and Evidence-Based Medicine, Faculty of Medicine, Universiti Malaya, 50603 Kuala Lumpur, Malaysia; 2 Hospital Serdang, 43000 Kajang, Selangor, Malaysia; 3 Centro Javeriano de Oncología, Hospital Universitario San Ignacio, Kra 7° # 40-62, Bogotá, Colombia; 4 Erasto Gaertner Hospital, PPGTS/Pontifícia Universidade Católica do Paraná, Curitiba, Brazil; 5 Regional Cancer Treatment Services, MidCentral District Health Board, 4410 New Zealand; 6 Institute of Cancer Policy, Global Oncology Group, School of Cancer Sciences, King’s College London, Strand, London, WC2R 2LS, United Kingdom

**Keywords:** contingency plan, emergency preparedness, cancer care, low- and middle-income countries, COVID-19

## Abstract

**Background:**

Many countries appear to be ill-prepared in their emergency responses towards the Corona Virus Disease 2019 (COVID-19) pandemic, particularly in managing chronic diseases such as cancer. We aimed to gain insight on the preparedness of health systems within low- and middle-income countries (LMICs) in maintaining delivery of cancer care amid the pandemic.

**Methods:**

We performed a rapid review of publications focusing on emergency contingency plans for cancer care during the pandemic in LMICs. An online desk research was conducted to identify relevant policy documents, guidelines or scientific publications.

**Results:**

Very few LMICs had readily accessible documents to ensure continuity in delivery of cancer care during the pandemic. A majority of publications were focused on delivery of cancer treatment whereas early detection, diagnosis and delivery of supportive and survivorship care received very little attention. Far fewer of the published guidelines appear to have been formulated at the national level by governmental agencies. A vast majority of publications constituted consensus guidelines from professional societies, followed by sharing of best practices from local institutions. Overall, three main strategies have been recommended to maintain delivery of cancer care amid the pandemic in LMICs: 1) Modification of cancer treatment regimens, 2) Changes in methods of administration of curative and supportive cancer care and 3) Implementation of generic measures to reduce the risk of COVID-19 infection in healthcare settings.

**Conclusion:**

All LMICs should consider collating best practices from the current pandemic and translating them into an explicit cancer preparedness plan, which can be escalated during future disasters.

## Background

The World Health Organization declared the novel Corona Virus Disease (COVID-19) outbreak a pandemic on 11 March 2020. As of 1 September 2021, almost 220 million confirmed COVID-19 cases have been reported globally, with close to 4.5 million deaths. Strikingly, the battle against this unprecedented global health security crisis has brought to light the systemic failures of governments and health systems in addressing long-standing health disparities between and within countries. Recognising that COVID-19 intersects with an array of non-communicable diseases including cancer, there have been calls to view the devastations brought about by the pandemic from a synergistic epidemic (syndemic) perspective [1]. Specifically, the clustering of COVID-19 and cancer within specific populations amid a backdrop of social and economic disparities is expected to exacerbate the adverse effects of each of these diseases [2].

It is, therefore, conceivable that the impact of the COVID-19 syndemic will be worse in low- and middle-income countries (LMICs), where patients living with cancer are more likely to have taken a bigger hit. In these settings, the healthcare systems that are already stretched due to limitation of resources may have been forced to adopt further priority-setting exercises in delivering cancer care to accommodate the delivery of urgent COVID-19 care. Such measures may include redeployment of the oncology workforce, reducing availability of hospital beds for cancer patients, rerouting of oncology patients to other centres to receive cancer care and rationing use of scarce medical resources. This notion is corroborated by reports that since have emerged from LMICs, which showed that with the surge in number of COVID-19 cases in these settings, cancer surgeries have been scaled down, clinic hours were shortened, radiology services were curtailed, systemic anticancer treatment and radiotherapy administration were prioritised based on treatment benefits and delivery of home-based palliative care were almost halted [3–5].

An expensive lesson that we are learning from the COVID-19 pandemic is that the rise in prevalence of cancer in the LMICs when combined with public health failures renders the affected populations more vulnerable to health emergencies. Based on the definitions by the Emergency Events Database (EM-DAT), the COVID-19 pandemic may be classified as a biological disaster [6]. As emergency preparedness responses after a disaster have been predominantly reactive and traditionally centred around providing basic necessities, managing injured and displaced victims, as well as treating infectious diseases and acute conditions [7], management of chronic diseases amid disasters has been suboptimal even in the high-income countries (HICs) [8]. For instance, it was previously reported that following Hurricane Katrina in the USA and the Tōhoku Earthquake in Japan, there were substantial loss of medical records, and disruption in surgical and adjuvant therapy services [9].

Central to this discussion is that in the absence of explicit guidelines, which are engineered to kick in during disasters, healthcare systems will be forced to redirect resources to the management of acute medical conditions based on morally arbitrary and reactive realities. In rooting for a holistic cancer policy that leaves no one behind including during disaster situations, we aimed to gain insight on the preparedness of health systems within LMICs in maintaining delivery of cancer care amid the COVID-19 pandemic.

## Methods

### Search strategy

Due to time and resource constraints, a targeted search method was employed rather than a traditional systematic review. Keyword searches were conducted using PubMed to identify relevant policy documents, clinical practice guidelines or scientific publications within LMICs. The search terms used were the names of the LMICs, followed by ‘COVID-19’ combined with ‘cancer care’, and ‘guidelines’, or ‘recommendations’ or ‘preparedness’. The list of LMICs was per World Bank income groups. Besides PubMed, Google Scholar was also used whereby up to the first 20 hits for every country were identified. Authors also searched for relevant information from the webpages of the Ministries of Health of the above countries. Scientific evidence was prioritised where available, but policy-relevant evidence was also drawn upon.

The criteria for inclusion were articles or reports from expert panel discussions or professional cancer societies, written in English or the native languages of the countries, with no restriction on cancer types, treatment modality and publication date. Exclusion criteria were any articles solely describing institutional experiences and impacts on cancer care without explicit recommendations.

**Figure 1.**
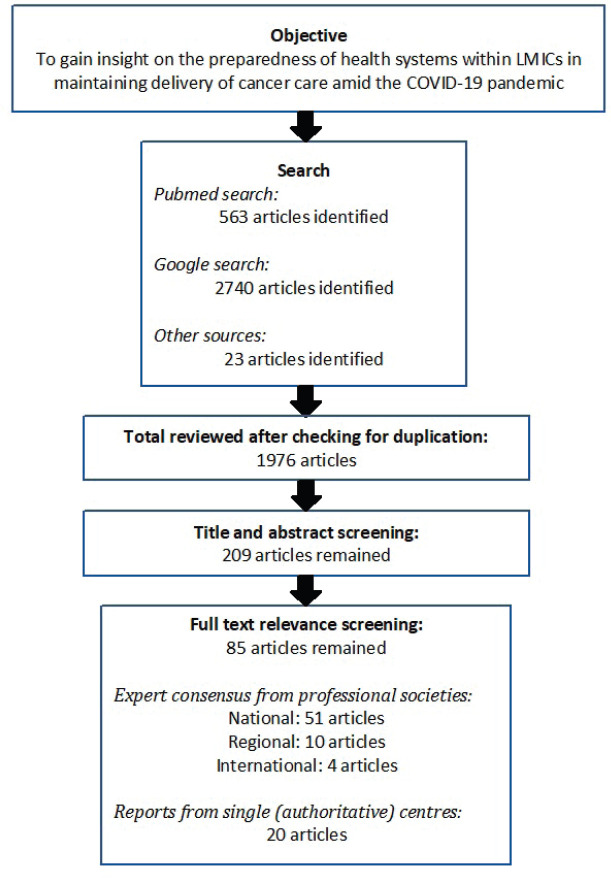
Search flow diagram.

Efforts were made to identify unpublished documents through personal communication with cancer experts from the region. The reference lists of key articles were also hand searched to identify additional literature that may be relevant. The desk search took about 10 weeks and the last search was conducted on 31 July 2021. The search flow is shown in Figure 1.

### Selection process

Authors from Latin America (RM, MZ) searched for articles in Spanish and Portuguese while multilingual authors from Asia (NBP, SP, YY) searched for articles in Asia. The search results from different sources were merged and removed for duplicates. Following this, NBP, SP, YY, RM and MZ identified eligible studies by screening titles and abstracts, and further retrieved the full text to assess for relevance; any disagreement was resolved through discussion. From the finalised list of literature, NBP, SP, YY, RM and MZ independently reviewed the contents and extracted the necessary data using a standardised data extraction table. Descriptive data analysis was conducted.

## Results

The search process for published literature identified 3,326 articles of potentially relevant articles. After the reviewers independently screened these titles and abstracts, 209 articles remained for full text screening. A total of 85 articles were identified after screening for relevancy. All of the included articles are summarised in Table 1 (10.6084/m9.figshare.17103029).

Our desk review of published literature revealed that less than 20% (25/135) of the LMICs had some form of locally formulated guidelines or recommendations to ensure continuity in delivery of cancer care during the COVID-19 pandemic. A vast majority of publications nonetheless constituted consensus guidelines issued by professional societies, followed by sharing of best practices by experts from local institutions. Notably, far fewer of the published guidelines appear to have been formulated at the national level by governmental agencies such as the ministries of health (top-down).

It was observed that most publications were from the Central or South American countries, followed by the LMICs from Asia, Africa and Europe. Strikingly, almost all the guidelines that were initiated top-down were from the Latin American nations including Argentina [10, 11], Brazil [12], Ecuador [13], Mexico [14] and Peru [15]. In Latin America, general recommendations for oncology care or recommendations for transversal services such as surgery or radiotherapy were predominant. However, breast cancer and haematological malignancies also had a relevant representation in guideline development.

In Asia, various approaches were noted. While the Philippine Society of Medical Oncology had published separate consensus recommendations for management of eleven types of cancers during the COVID-19 pandemic [16], India had a number of guidelines drawn up by different groups for different types of cancers [17–19]. The National Cancer Grid of India meanwhile appears to be finalising a set of official guidelines for cancer management during the COVID-19 outbreak [20]. Published literature from the African region were generally scanty, with mostly covering institutional responses to COVID-19 [21–22]. Country-specific recommendations also appear limited in the LMICs in Europe. Nonetheless, the European Parliament under a special committee had conducted a stakeholder’s public consultation involving various agencies including from its low- and middle-income member countries, and outlined a set of short-term and sustainable solutions to ensure continuity of cancer services during the pandemic, and in the event of future crises [23].

A majority of publications were focused on adjustments in delivery of cancer treatment as well as on COVID-19 preventive measures. Comparatively, recommendations on maintaining cancer diagnostic services were covered far less frequently, as was also the case for delivery of survivorship care. The domains within the cancer care continuum that received the least attention included early detection, supportive/palliative care and cancer research. Noteworthily, none of the guidelines or recommendations that were issued for the LMICs covered cancer registration.

Overall, three main strategies were implemented/recommended in the LMICs to maintain delivery of cancer care amid the COVID-19 pandemic. Firstly, modification of cancer treatment regimen, i.e. changing administration of chemotherapies from intravenous to oral routes, shortening of treatment regimens and delaying some cancer surgeries [4, 24, 25]. Secondly, changes in the methods or patterns of administration of cancer care, i.e. teleconsultation and supportive care provision to address nutritional needs, pain and wound care via short text messages, video/phone calls and web-based platforms, shifting cancer surgeries from public facilities to private or non-COVID-19 hospitals, decentralisation of chemotherapy to home-based administration, provision of patient navigation services to maintain access to clinics for medication supplies and also extension of prescription for low-risk treatments such as hormone therapy [26–28]. The third strategy comprised generic measures to reduce the risk of COVID-19 infection in the healthcare setting, i.e. solutions to enable physical distancing and reduce crowding, as well as ensuring personal and environmental hygiene [24–29].

## Discussion

Our rapid review shows that a majority of the presently available guidelines for cancer care in LMICs during the pandemic were formulated by professional societies, with the local cancer referral centres also playing an important role. It is however felt that governmental agencies such as the ministries of health should be leading the way in LMICs. This is based on the premise that a contingency plan, which is initiated top-down may confer more benefits to the health systems in resource-limited settings as it will conceivably be accompanied by governmental commitment to provide resources to sustain operations, besides facilitating standardisation of cancer care delivery at the national level.

It was also found that many of the guidelines for cancer care during disaster situations in LMICs seem to be heavily focused on delivery of cancer treatment, with far less attention given to other important areas such as early detection, diagnosis, and delivery of supportive and survivorship care. Nonetheless, it is acknowledged that contingency plans for delivery of some tranversal services such as surgery and palliative care are indeed available in LMICs, although not targeted to cancer alone [30, 31]. Remarkably, cancer registration seem to have been missed out from the emergency response planning in LMICs. In a survey by the International Agency for Research on Cancer in November 2020, half of the Caribbean registries reported having been negatively affected by the COVID-19 pandemic, particularly in terms of data collection [32]. It must be remembered that failures in planning to sustain cancer registration amid the pandemic could further undermine cancer responses and resource allocation in the LMICs, which are already struggling with high burden of the disease pre-pandemic. Nonetheless, our study findings must be considered together with the fact that many of the official cancer guidelines that were developed by the HICs in response to the COVID-19 pandemic, are also heavily focussed on administration of anti-cancer therapies [33–36].

Moving forward, the LMICs should consider collating best local practices from the current pandemic and translating them into an explicit cancer preparedness plan, which can be escalated during future outbreaks or disasters. Silver linings in cancer care that have emerged including cessation of low value care, digital communication and decentralisation of cancer care [37] should not be forgotten. Importantly, formulators should be wary of the stark imbalances in the emphasis on various parts of the cancer continuum when formulating their emergency preparedness plans for the LMICs. Areas including early detection, cancer diagnosis, supportive/palliative care, survivorship care, as well as cancer registration should not be overlooked during disaster situations.

While it is laudable that a number of international professional societies have developed cancer care guidelines for consumption during the pandemic in the LMICs [38–41], it is felt that guidelines that have been co-designed with key cancer stakeholders from the respective countries are more likely to be applicable, and also successful in terms of uptake in the local settings [42]. We, therefore, call for the efforts from the cancer fraternity within each country to identify and adapt relevant guidelines by enriching them with their own real-world experiences from the pandemic. The local health system’s strengths and limitations should also be taken into account as there is no one-size-fits-all. While it is proposed that such a document must be holistic and encompass all parts of the cancer care continuum, at the least, it should cover three aspects: (1) how to change treatment regimens while balancing risks and benefits, (2) how to adapt delivery of cancer care to maintain access and continuity and (3) integration of general measures related to the specific challenges of the emergency.

There are several limitations in the current review. Firstly, we did not evaluate the quality of the publications that were included in the present work. We also acknowledge that some of the literature may have been missed. In some countries, for instance, the national guidelines may be perceived as ‘internal documents’ and, therefore, may not be accessible online. Nonetheless, this only serves to underscore the point that guidelines or emergency preparedness plans for cancer care in LMICs should be made available in the public domain to promote the spirit of sharing of best practices within and between countries.

## Conclusion

The global healthcare community has learned from the COVID-19 disaster that lack of preparedness is the biggest problem, and issues surrounding management of patients with chronic illnesses including cancer are somewhat similar in LMICs or HICs. To this end, the member states of the United Nations during its general assembly on 11 September 2020 had agreed to put the Sendai Framework for Disaster Risk Reduction: 2015–2030 at the centre of its COVID-19 recovery and rehabilitation policy. The Sendai Framework clearly states that in case of a disaster, patients with chronic conditions must be considered in all policy and plans, ensuring that they have access to lifesaving services [43].

One of the many key lessons for cancer control from the current pandemic as such is to invest in the preparedness of healthcare systems to provide timely medical response without compromising the quality of cancer care. Too many health systems were forced to decrease or shut down routine non-COVID care which has negatively impacted patient outcomes through delays in both cancer diagnosis and management [44]. All countries should also work towards integrating emergency preparedness plan for cancer care as a specific domain into their existing national cancer control strategies to mitigate the challenges imposed by disasters. At the same time, we would like to remind that any such adoptions into national policies, while desirable, warrants careful consideration that is based on sound scientific evidence. Advocating the basic principle of medicine – ‘Primum non nocere’, which means, first do no harm, it is recommended that any established national contingency plans for delivery of cancer care are routinely evaluated by key cancer stakeholders and adapted to ensure that they remain effective, safe and relevant in the local context, within national health security preparedness and planning.

## Conflicts of interest

All authors have no potential conflicts of interest to declare.

## Funding

There was no funding support for this study.

## Authors’ contributions

Conceptualisation: Nirmala Bhoo-Pathy, Soo-Peng Teoh

Methodology: Nirmala Bhoo-Pathy, Soo-Peng Teoh, Yee-Yin Hoo

Writing – original draft: Nirmala Bhoo-Pathy, Soo-Peng Teoh, Yee-Yin Hoo

Writing – review & editing: Nirmala Bhoo-Pathy, Soo-Peng Teoh, Yee-Yin Hoo, Raul Murillo, María Zuluaga, Audrey Tsunoda, Dorothy Lombe, Richard Sullivan

Final approval: Nirmala Bhoo-Pathy, Soo-Peng Teoh, Yee-Yin Hoo, Raul Murillo, María Zuluaga, Audrey Tsunoda, Dorothy Lombe, Richard Sullivan
